# Photosynthetic Diffusional Constraints Affect Yield in Drought Stressed Rice Cultivars during Flowering

**DOI:** 10.1371/journal.pone.0109054

**Published:** 2014-10-02

**Authors:** Marco Lauteri, Matthew Haworth, Rachid Serraj, Maria Cristina Monteverdi, Mauro Centritto

**Affiliations:** 1 Institute of Agro-Environmental and Forest Biology, National Research Council, Porano, Italy; 2 Trees and Timber Institute (IVALSA - Istituto per la valorizzazione del legno e delle specie arboree), National Research Council, Florence, Italy; 3 Consultative Group on International Agricultural Research, Independent Science and Partnership Council Secretariat, Food and Agriculture Organisation of the United Nations, Rome, Italy; 4 International Rice Research Institute, Los Banos, Philippines; 5 Forestry Research Centre, National Research Council, Arezzo, Italy; Universidade Federal de Vicosa, Brazil

## Abstract

Global production of rice (*Oryza sativa*) grain is limited by water availability and the low ‘leaf-level’ photosynthetic capacity of many cultivars. *Oryza sativa* is extremely susceptible to water-deficits; therefore, predicted increases in the frequency and duration of drought events, combined with future rises in global temperatures and food demand, necessitate the development of more productive and drought tolerant cultivars. We investigated the underlying physiological, isotopic and morphological responses to water-deficit in seven common varieties of *O. sativa*, subjected to prolonged drought of varying intensities, for phenotyping purposes in open field conditions. Significant variation was observed in leaf-level photosynthesis rates (*A*) under both water treatments. Yield and *A* were influenced by the conductance of the mesophyll layer to CO_2_ (*g*_m_) and not by stomatal conductance (*g*_s_). Mesophyll conductance declined during drought to differing extents among the cultivars; those varieties that maintained *g*_m_ during water-deficit sustained *A* and yield to a greater extent. However, the variety with the highest *g*_m_ and yield under well-watered conditions (IR55419-04) was distinct from the most effective cultivar under drought (Vandana). Mesophyll conductance most effectively characterises the photosynthetic capacity and yield of *O. sativa* cultivars under both well-watered and water-deficit conditions; however, the desired attributes of high *g*_m_ during optimal growth conditions and the capacity for *g*_m_ to remain constant during water-deficit may be mutually exclusive. Nonetheless, future genetic and physiological studies aimed at enhancing *O. sativa* yield and drought stress tolerance should investigate the biochemistry and morphology of the interface between the sub-stomatal pore and mesophyll layer.

## Introduction

Global production of rice (*Oryza sativa*) is limited by the availability of freshwater [1] and the tolerance of rice cultivars to drought stress [2]. Nonetheless, *O. sativa* is cultivated in a diverse range of climates and habitats, with lowland varieties generally considered to be more productive, but less tolerant of drought, than their upland counterparts [3]. Selective breeding of *O. sativa* varieties has increased the grain production of individual plants by 70% since 1965 [4]. However, these increases in productivity have been restricted by the comparatively low ‘leaf-level’ photosynthetic capacity of *O. sativa* relative to other C3 crops [5], [6]. Phenotypic screening of the photosynthetic and gas-exchange characteristics of *O. sativa* varieties under water deficit conditions may permit the identification of attributes that confer both drought tolerance and high productivity [7], [8].

Yield in varieties of *O. sativa* are closely related to photosynthesis under both well-watered and drought conditions [7], [9]. The uptake of CO_2_ for photosynthesis involves two major resistance steps at the stomata and at the surface of the mesophyll layer [10], [11]. Stomatal (*g*_s_) and mesophyll conductance (*g*_m_) to CO_2_ often decrease in unison in response to environmental stresses [7], [12]–[14]. Upland and lowland *O. sativa* cultivars exposed to a soil moisture gradient ranging from high water-availability to severe water-deficit exhibited identical reductions in both *g*_s_ and *g*_m_ that correlated with the rate of photosynthesis (*A*) and yield. Most notably, while there was no observable difference in *A*, *g*_s_ or *g*_m_ response to drought between the upland and the lowland varieties, those cultivars that exhibited the highest values of total conductance to CO_2_ (*g*_t_: where *g*_t_  =  [*g*_s_ * *g*_m_]_/_[*g*_s_ + *g*_m_]) supported higher *A* and yield under all levels of water availability [7]. Those *O. sativa* varieties with higher capacities for CO_2_ uptake are therefore the most likely to offer the greatest potential improvement of leaf-level photosynthesis towards achieving enhanced yields [9], [15], [16]. However, an increase in the capacity for leaf gas exchange may be accompanied by reduced drought tolerance [17], [18].

These differences in the photosynthetic and leaf gas-exchange capacities of *O. sativa* varieties have been ascribed to genetic variation [9]. The quantitative trait loci responsible for leaf gas-exchange and photosynthetic parameters in *O. sativa* tend to be found in adjacent regions of the plant genome; indicative of the exertion of selective pressures favouring coordination and high heritability of photosynthetic behaviour [19], [20]. Therefore, effective screening and analysis of the photosynthetic and gas exchange characteristics of *O. sativa* varieties under stress conditions has the potential to identify functional traits associated with optimisation of water use efficiency (*WUE*) and the maintenance of yield and *A* under drought [21]. Rates of photosynthesis are not only determined by diffusive CO_2_-uptake, but also by the activity of ribulose-1,5-bisphosphate (RubisCO) [22]. However, exposure of thirteen hydroponically grown *O. sativa* varieties to physiological drought did not suggest that the capacity of RubisCO carboxylation (*V*c_max_) or the maximum rate of electron transport required for ribulose-1,5-bisphosphate regeneration (*J*_max_) were associated with *A* or grain yield. Rather, *A* and yield were largely determined by *g*_s_ and *g*_m_ setting the limits for diffusional CO_2_ uptake [9], consistent with field based studies [7]. Furthermore, the intrinsic water-use efficiency (*A*/*g*_s_) of the *O. sativa* varieties was closely related to the ratio of *g*_m_ to *g*_s_ [9], and this attribute showed the greatest variability between cultivars, and thus potential in terms of possible improvements in yield and growth through maximising *WUE* [5], [9], [21]. In theory, those varieties with high *g*_m_ to *g*_s_ ratios should be most suited to maintaining photosynthesis and yield under drought stress conditions, while high values of both *g*_m_ and *g*_s_ should be favourable for varieties grown under constant high water availability [21].

The observation that *A* and yield of *O. sativa* closely correlate to *g*_m_ [7], [9], [16] has led to a focus on the interface between the leaf mesophyll cells and internal sub-stomatal air space in the improvement of rice productivity [23]. Adachi et al. [16] crossed two varieties of *O. sativa* to produce a new cultivar with higher values of *g*_m_, which were associated with modifications of the mesophyll structure that increased the surface area available for CO_2_ uptake; thus demonstrating the potential for enhanced productivity through increased *g*_m_. In addition to morphological adaptations [15], differences in *g*_m_ between *O. sativa* varieties are also likely affected by biochemical factors [23] such as the abundance of cooporin proteins responsible for active transport of CO_2_ [24], [25] (Kaldenhoff, 2012). Furthermore, the abundance and activity of these proteins has been observed to decline under water-stress [26]. Exposure to drought is associated with declines in values of *A*, *g*_s_ and *g*_m_ [11], [27]. Sustained impairment of *A* following the alleviation of drought stress is commonly related with continued biochemical constraints to the uptake and assimilation of CO_2_ [28]. However, experimental evidence suggests that *O. sativa* does not display prolonged reductions in *A* following the cessation of drought, and that the observed constraints to *A* under water-stress are largely physical impediments to CO_2_ diffusion [7], [9], [29], [30]. This suggests that artificial selection of those *O. sativa* varieties with high *g*_m_ would enhance *A* while not negatively affecting drought tolerance or recovery.

Rice production is sensitive to water deficit at different developmental stages of plant growth. For example, early drought stress can affect leaf expansion [3], and later drought stress during flowering or grain-filling stages can disrupt grain formation and development [31], [32]. The photosynthetic and yield responses of seven *O. sativa* varieties (five upland and two lowland varieties chosen to represent genetic variability) were analysed in response to sustained drought. As different *O. sativa* varieties have different phenologies (for example Van forms flower inflorescences relatively earlier than Moro), through the use of a drip irrigation system it was possible to apply the stress at the same developmental stage for each variety. This has the advantage over a previous study into the drought tolerance of *O. sativa* varieties conducted at the IRRI in that all varieties experienced drought at the same phenological stage, therefore eliminating a crucial source of error whereby early flowering varieties outperformed late developing cultivars [cf. 7]. Mesophyll conductance to CO_2_ is of key interest to the productivity and drought tolerance of *O. sativa* [7], [16], however it is not possible to directly measure *g*_m_, and due to the uncertainties associated with its calculation [33] (particularly in the use of the variable J method under stress conditions) it is preferable to simultaneously use two independent methodologies in the estimation of *g*_m_ [34]. We therefore employed two independent methodologies in the estimation of *g*_m_: firstly, based upon the synchronous measurement of leaf gas exchange and chlorophyll fluorescence parameters (variable J), and secondly, the analysis of carbon isotope discrimination (Δ^13^C) in recently formed metabolites to assess their effectiveness in an analysis of *O. sativa* drought tolerance in the field. Further details of the variable J and ‘Δ^13^C of recently synthesised sugars’ methods of *g*_m_ analysis are presented in the Material and Methods section. This study aimed to: 1) assess the effect of *g*_s_ and *g*_m_ on *A* and yield in well-watered and drought conditions; 2) analyse differences in *g*_m_ calculation between the ‘variable J’ [35] and ‘Δ^13^C of recently synthesised sugars’ [15] methods, and; 3) identify *O. sativa* varieties that exhibit drought tolerance and/or high productivity, and may be suitable for further genomic/biochemical development to further enhance grain production.

## Materials and Methods

### Plant material and growth conditions

The experiment was conducted at the International Rice Research Institute Experimental Station at Los Banos, Philippines (14° 11′N 121° 15′E, 21 m above sea level) during the dry season (January to May). Rainfall was minimal, however, temperature and leaf to air vapour pressure difference (*VPD*) increased during the experimental period. The soil at the experimental site is a neutral Andaqueptic Haplaquol (pH = 6.5). Seven *O. sativa* cultivars were studied: Apo, IR55419-04 (IR55), IR64, IR71525-19-1-1 (IR71), Moroberekan (Moro), PSBRc80 (PS80) and Vandana (Van). Moroberekan failed to set or produce grains and seemed to suffer from disease and heat sensitivity, particularly under drought conditions and it was not possible to record physiological measurements for this variety under water deficit stress conditions. Seeds were sown in dry soil at a rate of 80 kg ha^−1^ in rows spaced ∼25 cm apart. Fertilisation with nitrogen was conducted on two occasions at application rates of 90 and 120 kg ha^−1^, alongside fertilisation of 30 kg ha^−1^ of phosphate and potassium. Weeds were controlled by the application of standard agricultural herbicides and manual picking. Pesticides were used to control insect pests; in particular stem borers.

Experimental plots for each *O. sativa* variety measured 3 m×2 m, with adjacent irrigated and drought-stressed treatments. The plots were arranged in a randomized block design. A sprinkler system was initially used to provide equal irrigation levels to all plants until flower (panicle) development, when the water deficit treatment was instigated. During the drought stress period, the well-watered treatments were irrigated by use of a drip irrigation system, with soil water potential maintained above -0.04 MPa (monitored using tensiometers placed at soil depths of 15 and 30 cm) – the level at which the more sensitive rice cultivars begin to exhibit a reduction in transpiration [36].

### Leaf gas exchange measurements

Leaf gas-exchange and fluorescence of plants grown under both well-watered treatment or stress conditions were simultaneously measured with a LI-6400-40 leaf chamber fluorometer (Li-Cor, Inc., Nebraska, USA) on flag leaves enclosed in a temperature, light and humidity controlled cuvette. The measurements were made *in situ* between 11.00 and 15.00, in saturating PPFD (1400 µmol m^−2^s^−1^), with relative humidity ranging between 45–55%, and a leaf temperature of 30°C. Instantaneous measurements of steady-state *A*, *g*_s_, internal [CO_2_] (*C*_i_) and the quantum yield of PSII in the light (ΔF/Fm') were made on six to 14 plants per treatment. Measurements of dark respiration (*R*_d_) were also made at ambient CO_2_ concentration in the dark on the same leaves. Instantaneous transpiration efficiency was calculated as the ratio of *A* to *g*_s_.

### Carbon isotope discrimination

Three of the same flag leaves used for *in situ* gas exchange measurements were collected in the evening, oven dried at 70°C until their weight remained constant and were then stored until soluble sugar extraction, following the protocol of Richter et al. [37]. Leaves were finely ground in liquid nitrogen and shaken for 60 min in water at room temperature. After centrifugation (15 min at 5000 g), the supernatant was sequentially mixed with cationic (Dowex-50) and anionic (Dowex-1) exchange resins. The residual solution of purified soluble sugars was freeze-dried and carbon isotope composition (δ^13^C) determined using a mass-spectrometer. Additional isotope ratio mass spectrometry analyses were performed on the pellet samples that remained after the extraction of soluble sugars. Samples of approximately 1 mg were used for δ^13^C analysis. Stable isotope ratios were determined using a continuous-flow triple-collector isotope ratio mass spectrometer (ISOPRIME, GV, Manchester, UK). Solid samples were quantitatively combusted in an elemental analyser (Model NA 1500, Carlo Erba, Milan, Italy) and CO_2_ was transferred in helium flow to the mass spectrometer. Calculations of carbon isotope discrimination (Δ^13^C) of solid samples were undertaken following the protocol of Farquhar et al. [38], assuming the carbon isotopic composition of CO_2_ in air (δ_air_) to be −8.0‰.

### Estimation of mesophyll conductance

Mesophyll conductance to CO_2_ diffusion is the inverse of the total resistance encountered in the transport of CO_2_ across the leaf mesophyll. Estimation of *g*_m_ is subject to a number of assumptions and sources of error, and it is preferable to employ two independent approaches in its calculation [34], [39]. In this study *g*_m_ was calculated using the ‘variable J’ and ‘Δ^13^C of recently synthesised sugars’ methods. The calculation of *g*_m_ using the variable J method is based on simultaneous measurements of gas-exchange and chlorophyll fluorescence parameters as described by Harley et al. [35] and Loreto et al. [40] (equations 1 and 2):
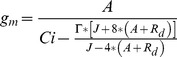
(1)where the electron transport rate (*J*_F_) is calculated from fluorescence [41]:



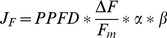
(2).

Where *F*_m_ is the fluorescence maximum and the partitioning factor (*β*) between photosystems I and II was considered to be 0.5 and leaf absorbance (*α*) was measured directly [42]. The variable *J* method is sensitive to the estimation of the CO_2_ compensation point to photorespiration (Γ*) and *R*_d_. For the results of this study to be directly comparable with those of Centritto et al. [7], the value of Γ* used in the gas exchange algorithm was calculated using the Rubisco specific factor for an annual herb [43], consistent with previously published *O. sativa* Γ* values [44]. As measurements were conducted under field conditions it was not feasible to produce robust measurements of respiration in the light (*R*_l_); respiration in the dark was therefore used in the calculation of gm, as *R*_l_ and *R*_d_ have been observed to be broadly consistent when recycling of CO_2_ is taken into consideration [45]. Measurements of *R*_d_ were performed at ambient [CO_2_] concentration in the dark on the same leaves by switching off the light in the leaf cuvette; when CO_2_ release from the leaf had become stable for approximately five to ten minutes this was recorded and considered to represent *R*_d_. To calibrate the values of *J* calculated by fluorescence (*J*_F_) and gas-exchange (*J*_A_), measurements were also conducted under non-photrespiratory conditions using 1% [O_2_] [35], [40]. Electron transport rate and *R*_d_ values are presented in Figures S1 and S2. Total conductance to CO_2_ (*g*_t_) was calculated as: *g*_t_  =  *g*_s_ * *g*_m_/(*g*_s_ + *g*_m_). The concentration of CO_2_ at the chloroplast site (*C*_c_) was then calculated from mesophyll conductance values [46].

The method described by Lauteri et al. [47] for ‘on-line’ determinations was adapted to calculate *g*_m_ by using the Δ^13^C of recently synthesized sugars [48]. Discrepancies between Δ^13^C of leaf soluble carbohydrates (Δ_obs_) and Δ^13^C expected on the basis of gas-exchange measurements (Δ_exp_), allow the estimation of *g*_m_ utilising the approach of Evans et al. [49] that was subsequently modified by Lloyd et al. [50]:
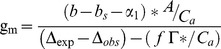
(3)


Where: *b* is the discrimination associated with carboxylation reactions, taken to be 27.5‰; *b*_s_ is the fractionation occurring when CO_2_ enters solutions (1.1‰ at 25°C); *a*_1_ is the fractionation during diffusion in water (0.7‰); *f* is the fractionation associated with photorespiration, and; *p*_a_ is the partial pressure of CO_2_ in air. Two *g*_m_ calculations were performed and compared, since two values were taken into account for the fractionation factor *f*: 7‰ [50] and 0‰ [48], [51]. The closest relationship between *g*_m_ estimates (R^2^ = 0.911) using the variable J and Δ^13^C of recently synthesized sugars methods in all varieties was found when using a *f* value of 0‰.

### Harvesting

Plants were harvested at physiological maturity by sampling 1 m of the 2 central rows of each plot. Data collected included biomass, plant height, anthesis date, tiller number, straw production, grain yield, and harvest index (*HI*).

### Statistical analysis

Analyses of variance were conducted using SAS to generate least squares means for each entry. Leaf gas exchange data were tested using a simple factorial ANOVA (three-way maximum interactions) and, where appropriate, the treatment means of leaf properties and gas exchange parameters were compared using a Tukey *post-hoc* test.

## Results

**Figure 1:**
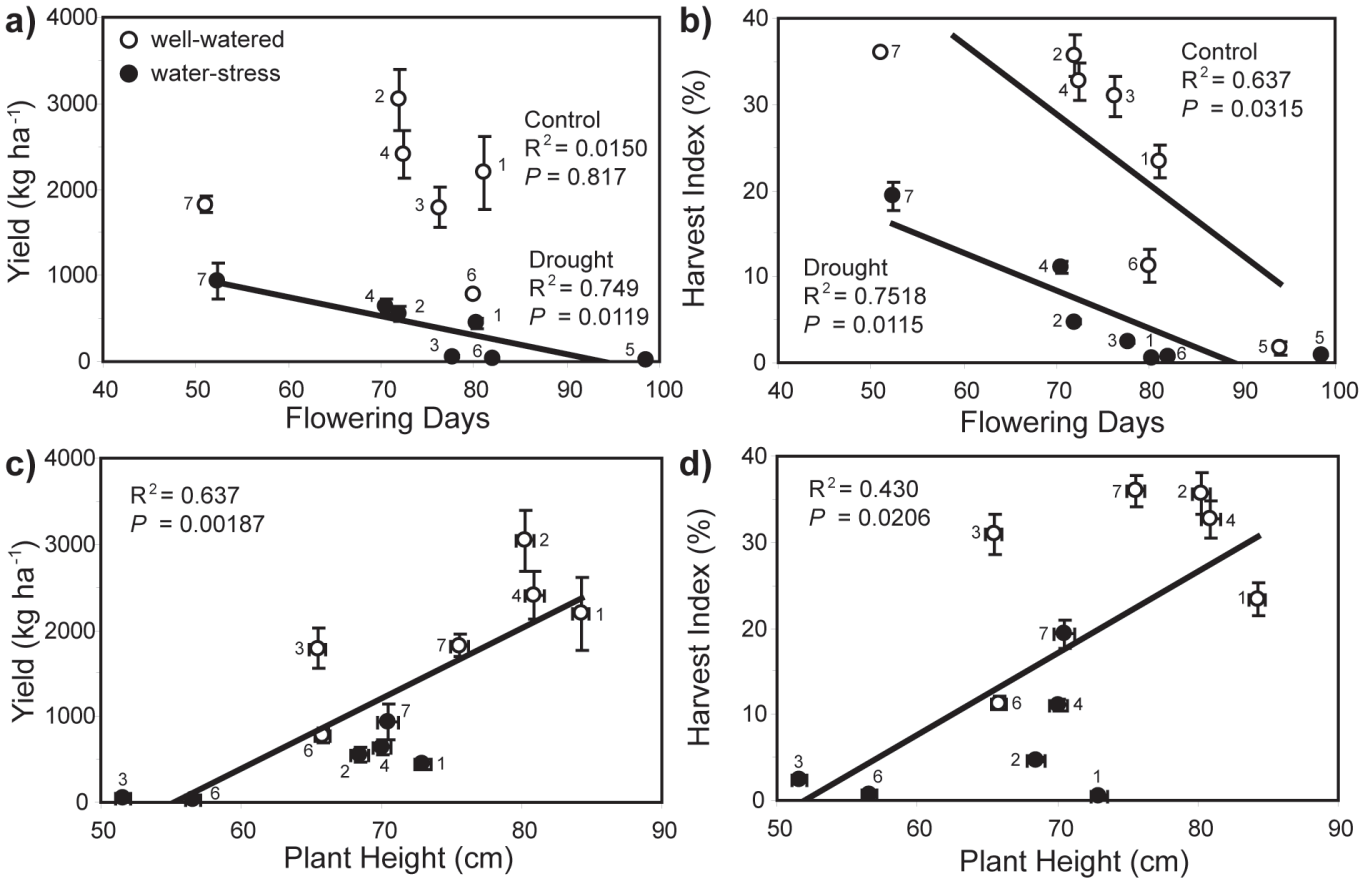
The performance of seven rice varieties under well-watered (open symbols) and drought conditions (closed symbols): a) relationship between yield and flowering days (period of time for 50% of plants to develop flowers) under full-water (linear regression: R^2^ = 0.0150; *F*_1,4_ = 0.0609; *P* = 0.817) and drought (linear regression: R^2^ = 0.749; *F*_1,5_ = 14.905; *P* = 0.0119); b) allometric relationship between yield and plant height (linear regression: R^2^ = 0.637; *F*_1,10_ = 17.536; *P* = 0.00187); c) relationship between harvest index (*HI*:dry weight of grain relative to dry total plant biomass) and flowering days under full-water (linear regression: R^2^ = 0.637; *F*_1,5_ = 8.760; *P* = 0.0315) and drought (linear regression: R^2^ = 0.752; *F*_1,5_ = 15.144; *P* = 0.0115), and; d) relationship between *HI* and plant height (linear regression: R^2^ = 0.430; *F*_1,10_ = 7.548; *P* = 0.0206). Error bars indicate one standard error either side of the mean. Numbers next to data points indicate *Oryza sativa* variety: 1 = Apo; 2 = IR55; 3 = IR64; 4 = IR71; 5 = Moro; 6 = PSBRc80; 7 = Van.

Drought stress induced reductions in yield, *A* and rates of gas-exchange across all the *O. sativa* varieties analysed in this study. The duration of time required for 50% of plants to develop flowers (flower days) did not correlate with yield in well-watered plants, but those varieties with a shorter flowering day period exhibited enhanced yield under drought stress (Fig. 1a). Varieties with shorter flowering days allocated a higher proportion of their total biomass to yield (harvest index: *HI*) under control and drought treatments (Fig. 1b). Positive allometric correlations between plant height and both yield (Fig. 1c) and *HI* (Fig. 1d) were observed, both under watered or drought conditions.

The carbon isotopic composition of the leaves of the *O. sativa* varieties became increasingly enriched in the heavier ^13^C isotope following water-stress, with the exception of IR64. Changes in the carbon isotopic composition of soluble sugars induced by the drought treatment were less consistent, with three varieties exhibiting increases and four decreases in the ratio of stable carbon isotopes (Table 1). Despite the lack of *genotype x water treatment* interaction, the varieties yielded differences in the ‘long-term’ isotopic signal of the bulk leaf material; some genotypes showed wide variation (up to −1.5‰ in pellets of Van) while others were more negligible (e.g. 0.2‰ in pellets of IR64). Drought caused also different variation in Δ^13^C of soluble sugars: up to −3.5‰ in PS80 but 0.2‰ in IR71. Under both well-watered and rainfed conditions, soluble sugars showed generally lower values of carbon isotopic discrimination when compared with the average Δ^13^C values of the flag leaf pellets. Significant differences were observed in the carbon isotopic discrimination values of of leaf pellets between varieties, however this was not replicated in the analysis of soluble sugars (Table 1).

**Table 1:** Carbon isotope discrimination (‰) of the flag leaf pellet (bulk) and soluble sugars of seven rice cultivars grown under drought and well-watered conditions in ‰.

Genotype	Habitat	Bulk Leaf Δ^13^C (‰)			Sugars Δ^13^C (‰)		
		Well-watered	Drought	Mean	Well-watered	Drought	Mean
Apo	U	21.6±0.9	20.6±0.3	21.0 ab	18.3±1.3	18.7±0.6	18.5 a
IR55	U	21.4±0.5	20.8±0.0	21.1 a	18.2±0.5	16.2±0.9	17.2 a
IR64	L	20.9±0.2	21.1±0.5	21.0 ab	18.5±0.6	16.6±0.8	17.5 a
IR71	U	21.1±0.2	20.7±0.5	20.9 ab	17.3±1.3	17.5±0.7	17.4 a
Moro	U	19.9±0.4	19.7±0.2	19.8 b	18.3±0.6	19.5±0.2	18.9 a
PS80	L	21.4±0.4	21.2±0.4	21.2 a	19.3±0.3	15.8±0.2	17.2 a
Van	U	21.5±0.3	20.0±0.5	20.7 b	18.1±1.5	15.8±0.4	17.0 a
Mean		21.1 a	20.6 b		18.2 a	17.2 b	

Values indicate the mean of six plants; ± indicates the standard error of mean; different letters indicate significant differences (P≤0.05) among means according to an ANOVA.

**Figure 2:**
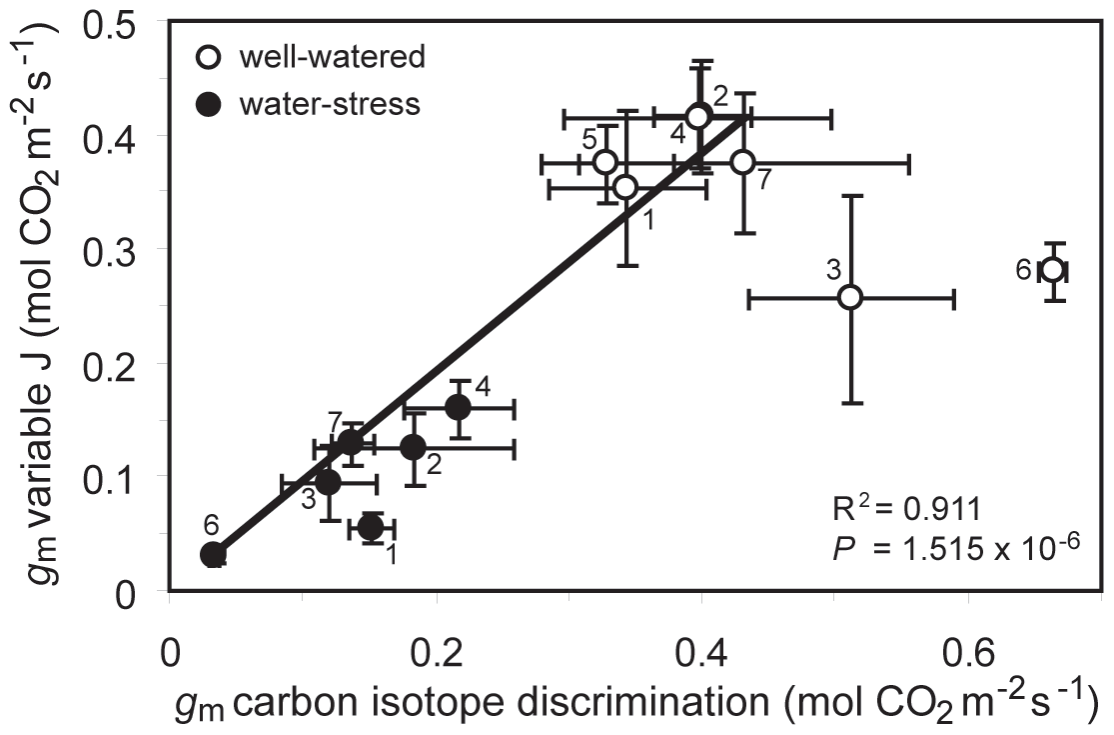
Comparison between the estimates of mesophyll conductance of CO_2_ (*g*_m_) obtained by applying two independent methods: the variable *J* method and the ‘δ13C of recently synthesised sugars’ method (linear regression: R^2^ = 0.932; *F*_1,9_ = 122.750; *P* = 1.515×10^−6^). Each data point represents the average value of three observations based upon the Δ13C of recently synthesised sugars and six to fourteen gas-exchange measurements utilising the variable J method. Error bars as in Figure 1. The regression line excludes the two data points on the right of the graph (IR64 and PS80) with anomalously high *g*_m_ derived from the δ13C of recently synthesised sugars. Numbers next to data points indicate *Oryza sativa* variety as in Figure 1.

**Figure 3:**
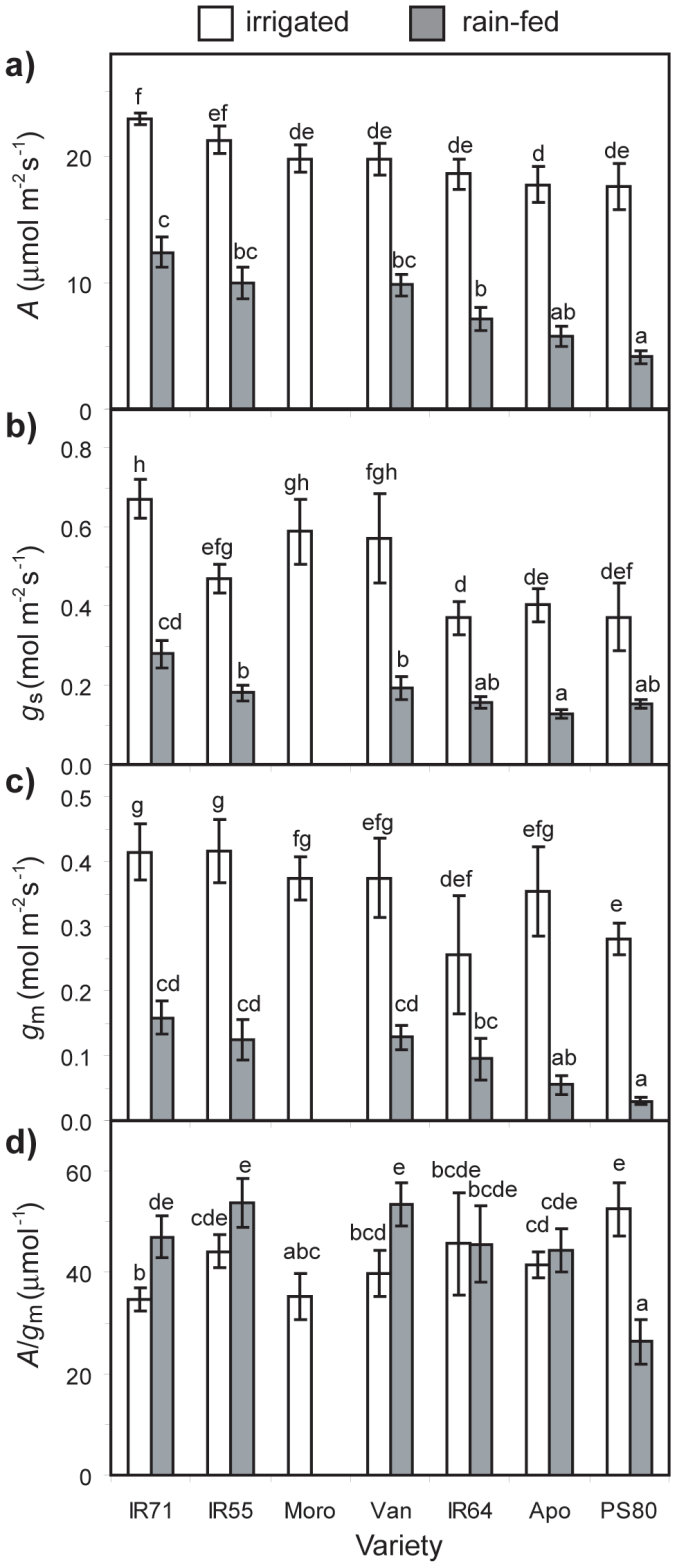
Measurements of (a) photosynthesis rate (*A*), (b) stomatal conductance (*g*_s_), (c) mesophyll conductance (*g*_m_), and (d) intrinsic transpiration efficiency (*A*/*g*_s_) in control and water-stressed leaves of the seven *Oryza sativa* genotypes. The measurements were made on the flag leaf in saturating PPFD (1400 µmol m^−2^s^−1^), with relative humidity ranging between 45–55%, and a leaf temperature of 30°C. Data are means of 4 to 7 plants per treatment. Error bars as in Figure 1. Different letters denote significant differences among means derived using a factorial ANOVA and Tukey *post-hoc* test.

**Figure 4:**
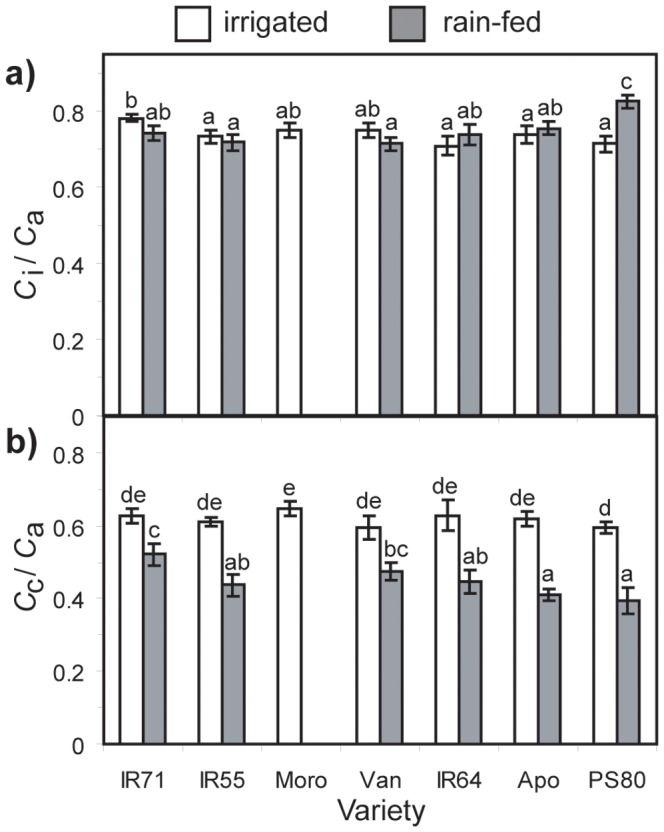
Measurements of (a) the intercellular [CO_2_] (*C*_i_) to the ambient [CO_2_] (*C*_a_) ratio (*C*_i_/*C*_a_), and (b) the chloroplastic [CO_2_] (*C*_c_) to the ambient [CO_2_] ratio (*C*_c_/*C*_a_) in control and water-stressed leaves of the seven *Oryza sativa* genotypes. The measurements were made on the flag leaf in saturating PPFD (1400 µmol m^−2^s^−1^), with relative humidity ranging between 45–55%, and a leaf temperature of 30°C. Data are means of 4 to 7 plants per treatment. Error bars as in Figure 1. Different letters denote significant differences among means derived using a factorial ANOVA and Tukey *post-hoc* test.

A strong positive correlation was observed between the two estimates of *g*_m_ in the *O. sativa* varieties grown under control and drought conditions (R^2^ = 0.930; P<0.01) (Fig. 2), excluding IR64 and PS80. Indeed, the lowland varieties IR64 and PS80 grown under well-watered conditions exhibited high values of *g*_m_ derived from the application of the isotopic methodology. It was therefore decided to utilise *g*_m_ measurements resulting from the variable J method in the subsequent analyses [an extended discussion of the contrasting merits of the approaches is given in 7]. Rates of *A*, *g*_s_ and *g*_m_ all showed consistent declines under water-stress conditions (Fig. 3). IR55 showed the highest yield and *g*_m_ under well-watered conditions. Variety IR71 exhibited the highest general values of *A*, *g*_s_ and *g*_m_ under both well-watered and drought conditions, while Apo and PS80 showed the lowest values of these photosynthesis and gas-exchange parameters under control and water-stress. The *A*/*g*_s_ ratio of the *O. sativa* varieties either increased (IR71; Van; IR55) or remained constant under drought stress in all varieties with the exception of PS80, where a 42.6% reduction was observed (Fig. 3d). The ratio of [CO_2_] within the substomatal pore (*C*_i_) to the external atmospheric [CO_2_] (*C*_a_) was largely unaffected by water-stress (Fig. 4a). In contrast, the ratio of the [CO_2_] within the chloroplast envelope (*C*_c_) to *C*_a_ was diminished in all *O. sativa* varieties during drought treatment (Fig. 4b), indicative of transport limitations of CO_2_ in the mesophyll for photosynthesis. *Oryza sativa* variety IR71 showed the smallest proportional reduction of 16.8% in the *C*_c_/*C*_a_ ratio, while Apo exhibited the largest reduction of 33.8%.

**Figure 5:**
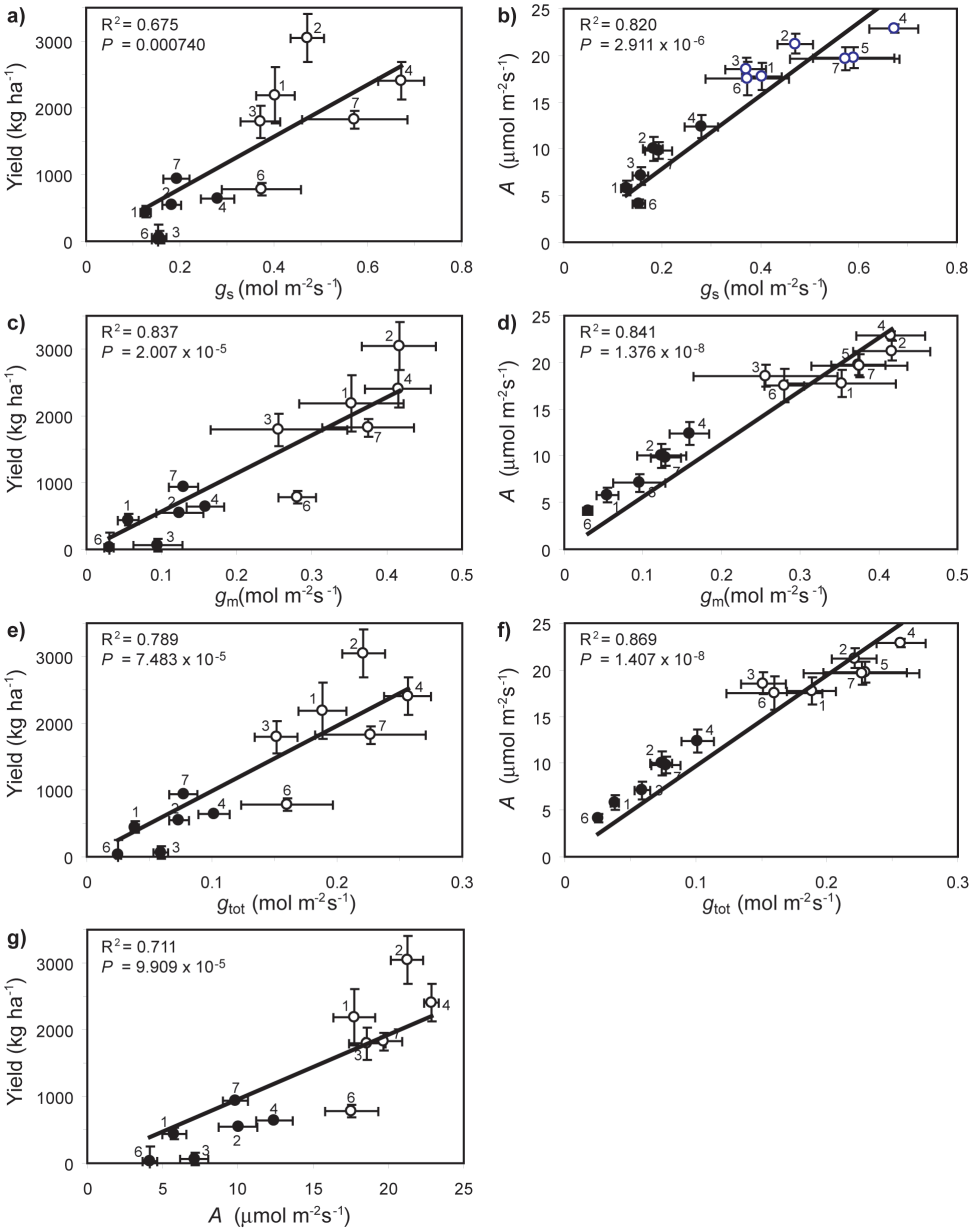
Interaction of diffusive conductance parameters to CO_2_ uptake with yield and photosynthesis (*A*) under well-watered (open symbols) and drought conditions (closed symbols): a) relationship between yield and stomatal conductance (*g*_s_) (linear regression: R^2^ = 0.696; *F*_1,10_ = 22.900; *P* = 0.000740); b) relationship between *A* and *g*_s_ (linear regression: R^2^ = 0.873; *F*_1,11_ = 75.721; *P* = 2.911×10^–6^); c) relationship between yield and mesophyll conductance (*g*_m_) (linear regression: R^2^ = 0.850; *F*_1,10_ = 56.611; *P* = 2.911×10^–5^); d) relationship between *A* and *g*_m_ (linear regression: R^2^ = 0.952; *F*_1,11_ = 217.071; *P* = 1.376×10^–8^); e) relationship between yield and total conductance (*g*_tot_) (linear regression: R^2^ = 0.806; *F*_1,10_ = 41.418; *P* = 7.483×10^–5^); f) relationship between *A* and *g*_tot_ (linear regression: R^2^ = 0.952; *F*_1,11_ = 216.173; *P* = 1.407×10^–8^), and; g) relationship between yield and *A* (linear regression: R^2^ = 0.795; *F*_1,10_ = 38.665; *P* = 9.909×10^–5^). Error bars as in Figure 1. Numbers next to data points indicate *Oryza sativa* variety as in Figure 1.

**Figure 6:**
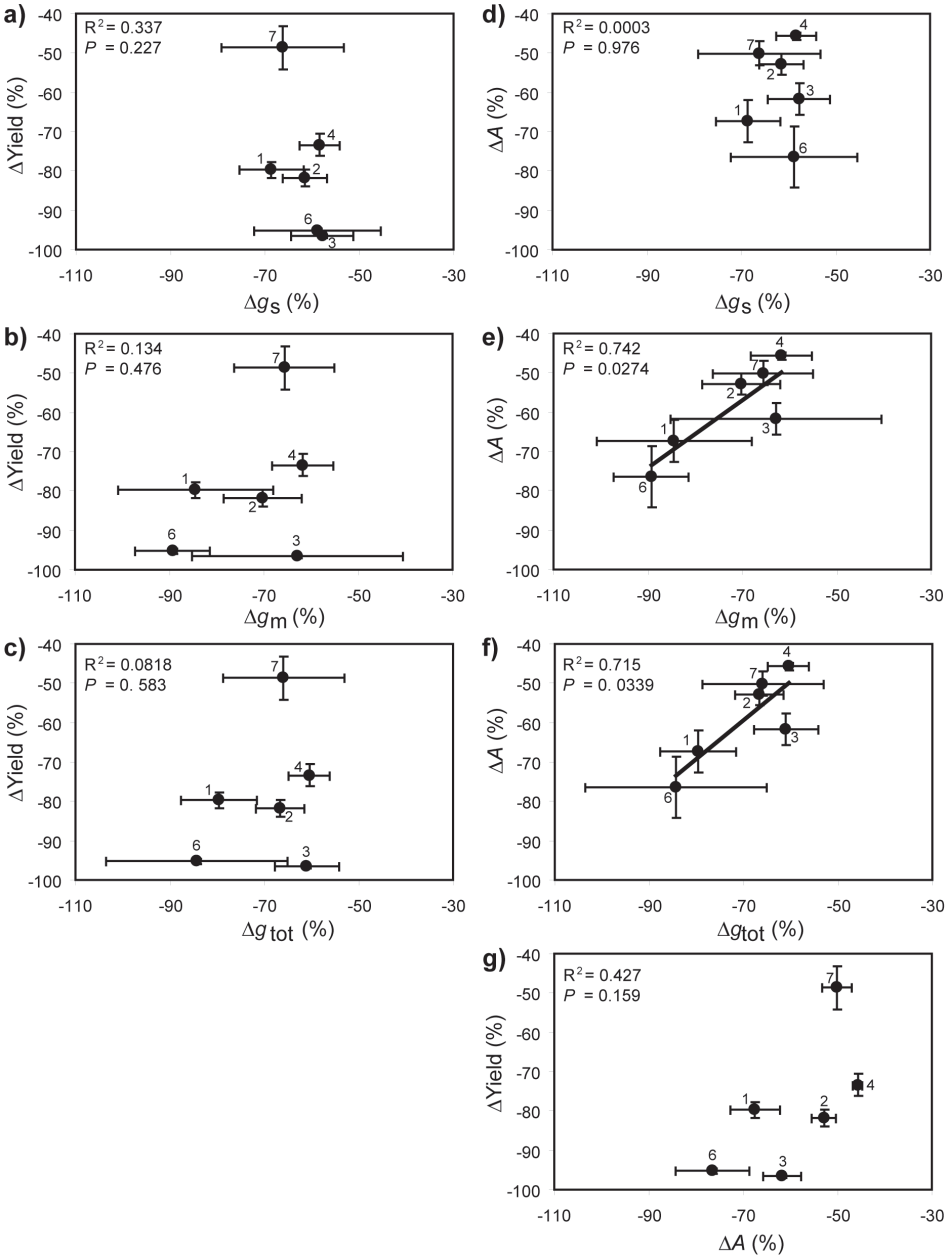
Changes in yield and photosynthesis in relation to modification of diffusive resistances to CO_2_ uptake following water-stress. Those varieties that experienced smaller reductions in parameters were more tolerant of drought. a) relationship between Δyield and Δ*g*_s_ (linear regression: R^2^ = 0.337; *F*_1,4_ = 2.032; *P* = 0.227); b) relationship between Δyield and Δ*g*_m_ (linear regression: R^2^ = 0.134; *F*_1,4_ = 0.618; *P* = 0.476); c) relationship between Δyield and Δ*g*_tot_ (linear regression: R^2^ = 0.0818; *F*_1,4_ = 0.356; *P* = 0.583); d) relationship between Δ*A* and Δ*g*_s_ (linear regression: R^2^ = 0.0003; *F*_1,4_ = 0.00106; *P* = 0.976); e) relationship between Δ*A* and Δ*g*_m_ (linear regression: R^2^ = 0.742; *F*_1,4_ = 11.527; *P* = 0.0274); f) relationship between Δ*A* and Δ*g*_tot_ (linear regression: R^2^ = 0.715; *F*_1,4_ = 10.042; *P* = 0.0339), and; g) relationship between Δyield and Δ*A* (linear regression: R^2^ = 0.427; *F*_1,4_ = 2.979; *P* = 0.159). Error bars as in Figure 1. Numbers next to data points indicate *Oryza sativa* variety as in Figure 1.

**Figure 7:**
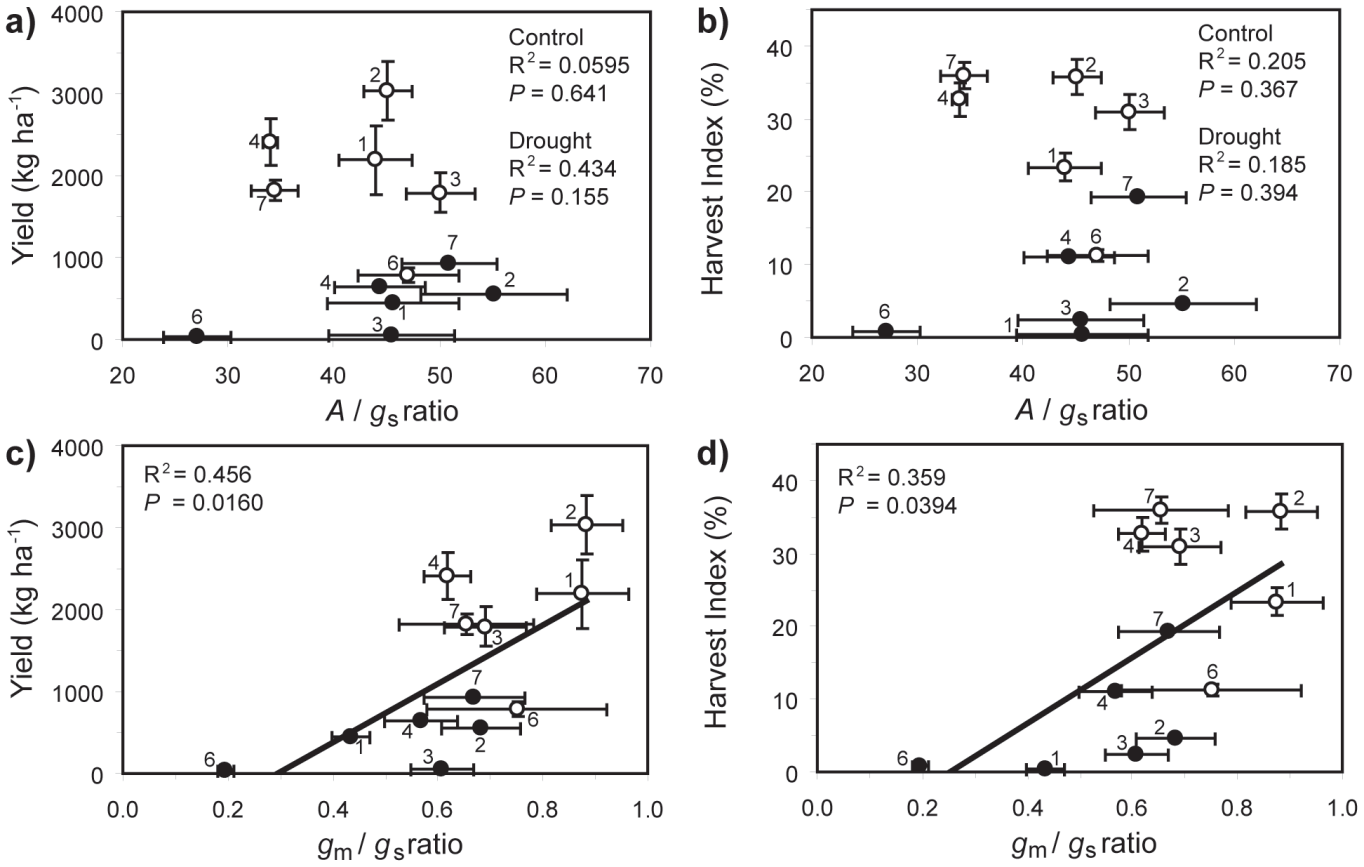
Interaction of yield and *A* with transpiration efficiency (*A*/*g*_s_) and the ratio of *g*_m_ to *g*_s_ in well-watered (open symbols) and drought conditions (closed symbols): a) relationship between yield and *A*/*g*_s_ under full (linear regression: R^2^ = 0.0595; *F*_1,4_ = 0.253; *P* = 0.641) and water-stressed (linear regression: R^2^ = 0.434; *F*_1,4_ = 3.072; *P* = 0.155) conditions; b) relationship between harvest index (*HI*) and *A*/*g*_s_ under full (linear regression: R^2^ = 0.205; *F*_1,4_ = 1.032; *P* = 0.367) and water-stressed (linear regression: R^2^ = 0.185; *F*_1,4_ = 0.909; *P* = 0.394) conditions; c) relationship between yield and *g*_m_:*g*_s_ (linear regression: R^2^ = 0.456; *F*_1,10_ = 8.379; *P* = 0.0160), and; d) relationship between *HI* and *g*_m_:*g*_s_ (linear regression: R^2^ = 0.359; *F*_1,10_ = 5.610; *P* = 0.0394). Error bars as in Figure 1. Numbers next to data points indicate *Oryza sativa* variety as in Figure 1.

Yield and *A* exhibited significant positive correlations with *g*_s_ (Fig. 5a and 5b). However, the positive correlations between yield and *A* with *g*_m_ (Fig. 5c and 5d) were stronger than those observed with *g*_s_ or total conductance (*g*_tot_); suggesting that *g*_m_ is the major determinant of yield and *A* under both well-watered and water-stressed conditions. The yield of the *O. sativa* varieties was strongly related to *A* under both control and drought treatments (Fig. 5g). In order to gauge the response of these diffusive limitations to CO_2_ transport and their effect on yield and *A*, the proportional change (Δ) in response to drought was calculated. The proportional reduction in yield (Δyield) was not related to Δ*g*_s_, Δ*g*_m_ or Δ*g*_tot_ (Fig. 6a; 6b; 6c). However, Δ*A* was significantly correlated to Δ*g*_m_ and Δ*g*_tot_. IR71 exhibited the highest levels of *A* and *g*_m_ (Fig. 3a and 3c), but also the lowest proportional reduction in *g*_m_ and *A* (Fig. 6d). The highest yields observed under control conditions were found in IR55 (3039.6±359.1 kg ha^−1^) and IR71 (2407.4±284.4 kg ha^−1^); under water-stress the yield of IR55 fell to 552.7±80.3 kg ha^−1^ and IR71 to 640.3±93.0 kg ha^−1^. The lowest Δyield reduction under drought conditions was observed in variety Van (Fig. 6), which also produced the highest yield under water-stress (935.3±206.0 kg ha^−1^). IR71 exhibited the second lowest Δyield and produced second largest yield during water-stress of the varieties analysed (Fig. 6g). The *A*/*g*_s_ ratio was not significantly related to either yield or *HI* under both well-watered or drought conditions (Fig. 7a and 7c). However, the *g*_m_:*g*_s_ ratio was significantly correlated with both yield and *HI* (Fig. 7b and 7d), suggesting that those *O. sativa* varieties with greater *g*_m_/*g*_s_ ratio will exhibit enhanced productivity under both well-watered and water-stressed conditions.

## Discussion

The seven *O. sativa* varieties exhibited a diverse range of morphological, isotopic, photosynthetic and leaf gas-exchange responses to drought stress. A number of factors determined the yield of the *O. sativa* cultivars analysed in this study. Despite water deficit being instigated at the same developmental stage for all varieties, phenology had an effect on yield under drought as rapidly developing plants maintained yield to a greater extent than slower developers (Fig. 1a) [52]. Despite the type and phenolgical timing of the drought stress being different to that utilised in the investigation of Centritto et al. [7], the effect on *A* and yield observed in the two studies are broadly consistent. Those plants that were able to accumulate biomass rapidly produced higher grain yields than those that accumulated biomass less efficiently under both well-watered and drought conditions. This may be due to allometric relationships between biomass and yield [53], or the positive influence of increased photosynthetic area on ‘whole-plant’ carbon uptake [54]. Leaf-level photosynthetic rates were also closely related to yield in all varieties under both well-watered and drought conditions (Fig. 5g). Photosynthesis rates were in turn determined by diffusive limitations to CO_2_-uptake (Fig. 5b; 5d; 5f). The most significant of these resistances to CO_2_-uptake in *O. sativa* occurs at the interface between the internal sub-stomatal air-space and the mesophyll layer [15], [55]. Consistent with other studies, *g*_m_ in *O. sativa* correlated most closely with *A* and yield (Fig. 5d) [7], [9], and represents a critical ‘rate-limiting’ step in the determination of grain productivity and assessment of the photosynthetic response to drought stress.

The ‘bulk’ carbon isotope discrimination values of the *O. sativa* varieties decreased following drought treatment; indicative of water-deficits over the life-span of the flag-leaf reducing discrimination against the heavier ^13^C isotope [56], [57]. However, this pattern of reduced carbon isotope discrimination is not apparent in the carbon isotopic composition of recently synthesised sugars (Table 1). These findings may reflect ‘shorter-term’ variations in *WUE* in the plants experiencing drought stress, a modification of the photosynthetic pathway affecting sugar metabolism [28] or the breakdown of previously stored carbohydrates with a different isotopic signature into more simple sugars over the course of the stress treatment [58], [59]. Differences in the diffusional uptake of CO_2_ likely affected carbon isotopic composition [60]; however, this was not apparent from our *g*_s_, *g*_m_ (variable J) and *g*_tot_ datasets (Fig. 5), suggesting that other factors affected carbon isotopic discrimination and possibly the effectiveness of the Δ13C of recently synthesised sugars method of estimating *g*_m_ in varieties IR64 and PS80 under control conditions (Fig. 2). It is noteworthy that under water-deficit conditions, *g*_m_ estimates based on the variable J method closely correlated with those derived from the Δ13C of recently synthesised sugars (Fig. 2). These findings do not support the suggestion that reductions in *C*_i_ may affect estimation of the total photosynthetic electron transport rate associated with enhanced photorespiration, thus rendering the variable J method unreliable in the investigation of *g*_m_ in plant responses to environmental stress [34].

Selective breeding has improved the productivity of *O. sativa* varieties through improved growth form and the increased proportional allocation of biomass as grain [4]; yet these increases in yield have been constrained by the relatively low leaf-level photosynthetic rate of *O. sativa* in comparison to other C3 crops [5], [61]. The results of this study indicate that under well-watered and water-deficit conditions, *A* is an important determinant of yield in *O. sativa* and is largely related to the rate of transport of CO_2_ across the mesophyll (Fig. 5d). Further improvements in *O. sativa* grain yield may likely be achieved through the enhancement of *g*_m_ to CO_2_ inducing enhanced *A* [6], [9], [16], [62]. Those *O. sativa* varieties that showed the greatest photosynthetic performance and yield under control conditions were not necessarily the most effective varieties under drought conditions. Varieties that maintained transport of CO_2_ during water-stress sustained *A* and yield to a greater extent than varieties that exhibited more pronounced diffusive limitations to photosynthetic CO_2_-uptake (Fig. 5). Drought stress induced a relatively consistent mean reduction in *g*_s_ in the *O. sativa* varieties (Fig. 3), yet the *C*_i_:*C*_a_ ratio was unaffected (Fig. 4), indicating that *g*_s_ was not limiting to *A* under water-deficit, or that the gas-exchange data were possibly biased by the occurrence of non-uniform stomatal closure over the leaf lamina and consequent overestimation of *C*_i_ [27], [63], [64]. However, the *C*_i_/*C*_a_ ratio in the *O. sativa* varieties was reduced by 16.8 to 33.8% after drought, consistent with the impairment of *A* through mesophyll diffusive limitations [7], [11], [12], [65], [66]. Mesophyll conductance to CO_2_ declined during the drought treatment, constraining *A* and growth (Fig. 5; 6). However, significant variability in the extent of reductions in *g*_m_ were observed in the *O. sativa* cultivars ranging from −61.7% (IR71) to −89.3% (PS80). Furthermore, those varieties that showed the smallest reductions in *g*_m_ also exhibited greater *A*; demonstrating that *g*_m_ largely determines *A* in *O. sativa* under differing degrees of water-deficit [7], [9]. These differences in the ability to sustain *g*_m_ values under drought stress are likely related to the maintenance of biochemical function in the mesophyll such as the activity of cooporin and carbonic anhydrase [eg. 25,67].

The conductance of the mesophyll layer to the transport of CO_2_ determined *A* and yield in the seven *O. sativa* varieties studied. Under conditions of water-stress it has been suggested that a low *g*_s_ (to reduce transpirative water-loss) and a high *g*_m_ (to maintain the uptake of CO_2_) are beneficial [11], [68]. However, in the case of *O. sativa*, yield and *HI* were only weakly related to the ratio of *g*_m_:*g*_s_ (R^2^<0.460) (Fig. 7). The reduction in *g*_s_ under water-deficit is consistent with active stomatal control in the optimisation of *WUE* [69], [70]; nevertheless, the lack of a strong correlation to the *g*_m_:*g*_s_ ratio may be indicative of the lack of a stomatal limitation to *A* under drought stress in the *O. sativa* varieties apparent in the *C*_i_:*C*_a_ ratios (Fig. 4a). The photosynthetic and yield responses of *O. sativa* under well-watered and drought conditions were more dependent upon *g*_m_ than *g*_s_ (Fig. 5b and 5d). This is consistent with earlier studies reporting that modification and improvement of *g*_m_ is more effective in the enhancement of *O. sativa* productivity [16] and likely drought tolerance [7], [9]. The strong dependence of *A* and yield on *g*_m_ is also evident in the lack of a relationship between *A*/*g*_s_ and yield or *HI* (Fig. 7a and 7c). This may suggest that stomatal control is either less effective [70], [71], or plays a reduced role, in respect to mesophyll limitations to CO_2_ in a drought intolerant crop such as *O. sativa* in comparison to crops adapted to more water-limited environments [72].

The disparity in *A* values observed between the cultivars raises the possibility of crossing different varieties to enhance leaf-level *A* and yield in subsequent generations [6], [8], [16]. Selection of drought tolerance on the basis of the ability of a particular genotype to maintain *g*_m_ function under drought stress would be viable [11], [73]. However, genetic sequencing and identification of quantitative trait loci permit the possibility of extracting genetic material from a *O. sativa* cultivar with a valuable characteristic (such as high *g*_m_ and/or stable *g*_m_ under water deficit) and in the creation of a new variety with a suite of desired attributes [17], [19], [74]–[76]. For example, from the results of this study it would be desirable to combine the high *g*_m_ and yield of IR55 under well-watered conditions with the capacity to sustain *g*_m_ under water-stress exhibited by IR71 and water-stressed yield of Van (Fig. 3). In this context it may be possible to phenotypically screen a large number of *O. sativa* genotypes to identify quantitative trait loci responsible for *g*_m_ values and *g*_m_ response to drought. Those varieties developed to be resistant to drought may be suited to more marginal land-types or in scenarios of insufficient water availability. Nonetheless, given the sporadic and unpredictable nature of sustained and persistent drought events it is unlikely that large numbers of farmers would select a drought tolerant variety such as Van over a more productive cultivar such as IR55, and thus limit their harvest to accommodate the risk of interruption to water supplies [73], [77]. An ideal *O. sativa* variety would therefore possess both values of *g*_m_ capable of sustaining high *A* and the ability to maintain *g*_m_ in the event of water-deficits [8], [78]. However, the results of this study suggest that those attributes may be mutually exclusive in the species analysed. Furthermore, no single parameter analysed in this study exclusively accounted for yield under either well-watered or drought conditions; therefore attempts to improve the productivity of *O. sativa* cultivars may also involve selection for a suite of characteristics that improve productivity under specific conditions of water availability. Nonetheless, future efforts to improve *O. sativa* productivity should focus on biochemical [eg. 25] and physical modification [eg. 16] of the interface between the mesophyll layer and sub-stomatal air-space to enhance CO_2_ uptake and *A*.

## Conclusions

Physiological analysis of *O. sativa* has great potential in identifying those photosynthetic and leaf gas-exchange characteristics that confer attributes such as high yield and tolerance to drought. The analysis of the seven *O. sativa* varieties indicates that under both well-watered and drought stressed conditions yield and *A* are determined by diffusional constraints to CO_2_ uptake and not by biochemical limitations. The varieties showed significant variation in leaf-level photosynthetic rates, symptomatic of the photosynthetic constraints to *O. sativa* production that have previously been recorded. Selection of those *O. sativa* genotypes that exhibit higher rates of leaf-level *A* would likely be beneficial for grain yields. Photosynthesis was most closely related to *g*_m_, and the tolerance of the *O. sativa* varieties to drought was largely determined by the ability to maintain *g*_m_ values under water-deficits. However, the highest *g*_m_ and yield under well-watered conditions (IR55) and the most pronounced ability to sustain *g*_m_ under water-deficit (IR71) were observed in different varieties. This may suggest that the desired attributes for enhanced yield during high water availability and the ability to maintain *g*_m_, and thus sustain grain production under water-deficits, may be mutually exclusive. Nonetheless, the results of studies such as this could make it possible through selective breeding, or the identification of a particular genetic expression associated with a desired attribute, to increase leaf-level photosynthetic rates and drought tolerance in the same variety of *O. sativa*. These findings indicate that the interface between the internal sub-stomatal air-space and the mesophyll layer offers the greatest potential in terms of possible modification to enhance the performance of *O. sativa* under optimal and stressed growth conditions in the future.

## Supporting Information

Figure S1 **Dark respiration (** ***R*** **_d_) values of seven** ***Oryza sativa*** **varieties under irrigated (white) and rain-fed (grey) conditions used in the calculation of** ***g*** **_m_** **using the variable J method.**
(PDF)

Figure S2 **Electron transport rate (ETR) values of seven *Oryza sativa* varieties under irrigated (white) and rain-fed (grey) conditions used in the calculation of *g*_m_ using the variable J method.**
(PDF)
