# Correction: Photosynthetic Diffusional Constraints Affect Yield in Drought Stressed Rice Cultivars during Flowering

**DOI:** 10.1371/journal.pone.0117631

**Published:** 2015-02-19

**Authors:** The PLOS ONE Staff

The units on the y-axis of Fig. 3d are incorrect; the correct units are μmol mol^-1^. Please see the corrected Fig. 3 here.

**Figure 3:**
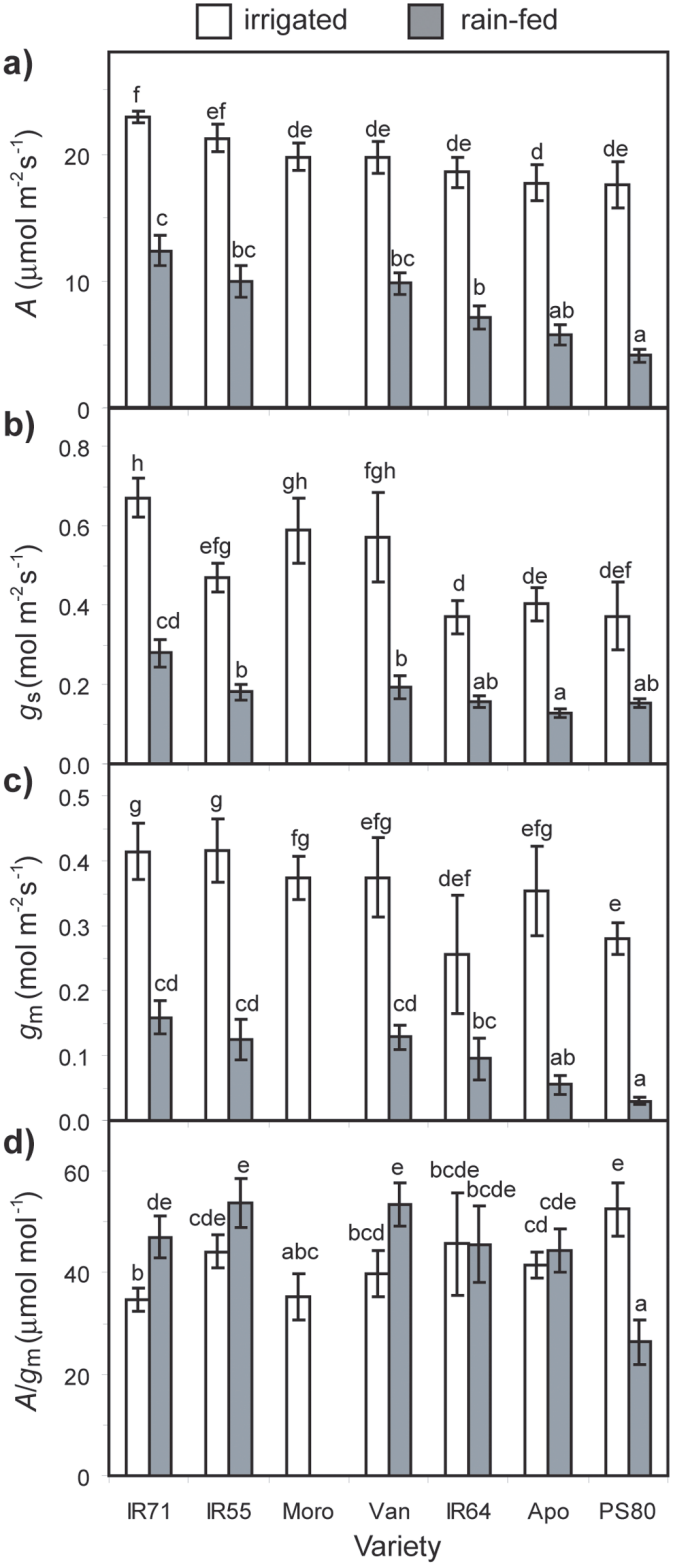
Measurements of (a) photosynthesis rate (*A*), (b) stomatal conductance (*g*_s_), (c) mesophyll conductance (*g*_m_), and (d) intrinsic transpiration efficiency (*A*/*g*_s_) in control and water-stressed leaves of the seven *Oryza sativa* genotypes. The measurements were made on the flag leaf in saturating PPFD (1400 μmol m^-2^s^-1^), with relative humidity ranging between 45–55%, and a leaf temperature of 30°C. Data are means of 4 to 7 plants per treatment. Error bars as in Figure 1. Different letters denote significant differences among means derived using a factorial ANOVA and Tukey *post-hoc* test.

The affiliation for Maria Cristina Monteverdi is incorrect. The correct affiliation is: Consiglio per la Ricerca e la Sperimentazione in Agricoltura, Centro di Ricerca per la Selvicoltura, Arezzo, Italy

There is an error in the funding statement. The correct statement is: This work was funded by: Ministero dell’Istruzione, dell’Università e della Ricerca of Italy: PRIN 2010–2011 PRO-ROOT, Progetto Premiale 2012 Aqua, CNR project Conoscenze Integrate per la Sostenibilità e l’Innovazione del Agroalimentare and a Marie Curie Intra European Fellowship (2010-275626)—HAWORTH—Evoutionary adaptation to atmospheric carbon dioxide, URL: http://ec.europa.eu/research/mariecurieactions/about-mca/actions/ief/index_en.htm. The funders had no role in study design, data collection and analysis, decision to publish, or preparation of the manuscript.

## References

[1] Lauteri M, Haworth M, Serraj R, Monteverdi MC, Centritto M (2014) Photosynthetic Diffusional Constraints Affect Yield in Drought Stressed Rice Cultivars during Flowering. PLoS ONE 9(10): e109054 doi: 10.1371/journal.pone.0109054 25275452 10.1371/journal.pone.0109054 PMC4183539

