# Supplementary material for: Photosynthetic Diffusional Constraints Affect Yield in Drought Stressed Rice Cultivars during Flowering
Source: PLoS One. 2014 Oct 2;9(10):e109054. doi: 10.1371/journal.pone.0109054 (PMC4183539; doi:10.1371/journal.pone.0109054)

S2: electron transport rate (ETR) values of seven *Oryza sativa* varieties under irrigated (white) and rain-fed (grey) conditions used in the calculation of g_m using the variable J method.

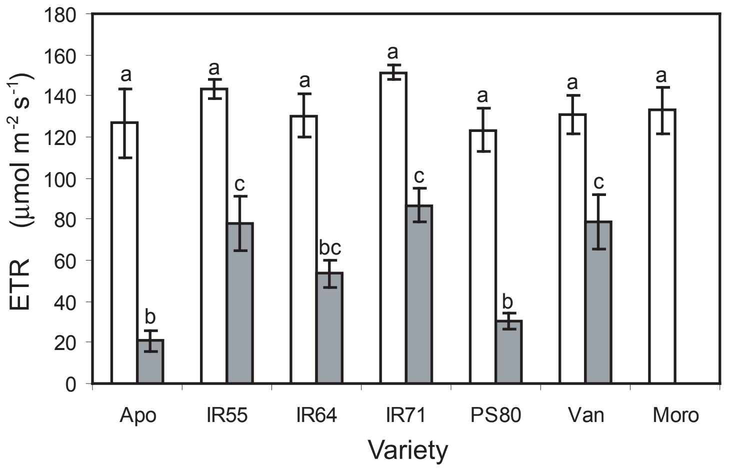

Supplement: Figure S2 — Electron transport rate (ETR) values of seven Oryza sativa varieties under irrigated (white) and rain-fed (grey) conditions used in the calculation of gm using the variable J method. (PDF) [file pone.0109054.s002.pdf]
